# 4-(Prop-2-yn-1-yl­oxy)benzaldehyde

**DOI:** 10.1107/S1600536812050866

**Published:** 2012-12-22

**Authors:** Ikue Doi, Tsunehisa Okuno

**Affiliations:** a Department of Material Science and Chemistry, Wakayama University, Sakaedani, Wakayama 640-8510, Japan

## Abstract

In the title mol­ecule, C_10_H_8_O_2_, all non-H atoms are essentailly coplanar (r.m.s. deviation = 0.0192 Å), indicating an effective conjugation of the carbonyl group, the benzene ring and the lone pair of the propyn­yloxy O atom. In the crystal, π–π stacking inter­actions [centroid–centroid distance = 3.5585 (15) Å] connect mol­ecules into inversion dimers which are linked by C*sp*—H⋯O=C hydrogen bonds, forming a ladder-like structure.

## Related literature

For related structures of 4-(prop-2-yn-1-yl­oxy)benzenes, see: Berscheid *et al.* (1992 ▶); Mohr *et al.* (2003 ▶); Nieger *et al.* (2004 ▶); Ranjith *et al.* (2010 ▶); Zhang *et al.* (2011 ▶).
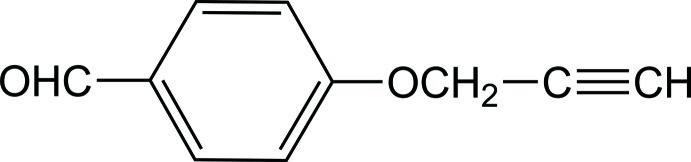


## Experimental

### 

#### Crystal data

C_10_H_8_O_2_
*M* *_r_* = 160.17
Monoclinic, 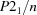

*a* = 7.906 (3) Å
*b* = 7.385 (2) Å
*c* = 14.036 (5) Å
β = 102.025 (5)°
*V* = 801.5 (5) Å^3^
*Z* = 4
Mo *K*α radiation
μ = 0.09 mm^−1^
*T* = 93 K
0.20 × 0.10 × 0.10 mm

#### Data collection

Rigaku Saturn724+ diffractometer
Absorption correction: numerical (*NUMABS*; Rigaku, 1999 ▶) *T*_min_ = 0.984, *T*_max_ = 0.991
6309 measured reflections
1832 independent reflections
1669 reflections with *F* ^2^ > 2σ(*F* ^2^)
*R*_int_ = 0.044

#### Refinement

*R*[*F* ^2^ > 2σ(*F* ^2^)] = 0.037
*wR*(*F* ^2^) = 0.101
*S* = 1.06
1831 reflections
141 parameters
H-atom parameters constrained
Δρ_max_ = 0.32 e Å^−3^
Δρ_min_ = −0.17 e Å^−3^

### 

Data collection: *CrystalClear* (Rigaku, 2008 ▶); cell refinement: *CrystalClear*; data reduction: *CrystalClear*; program(s) used to solve structure: *SHELXD* (Schneider & Sheldrick, 2002 ▶); program(s) used to refine structure: *SHELXL97* (Sheldrick, 2008 ▶); molecular graphics: *ORTEP-3* (Farrugia, 2012 ▶); software used to prepare material for publication: *CrystalStructure* (Rigaku, 2010 ▶).

## Supplementary Material

Crystal structure: contains datablock(s) global, I. DOI: 10.1107/S1600536812050866/lh5571sup1.cif

Structure factors: contains datablock(s) I. DOI: 10.1107/S1600536812050866/lh5571Isup2.hkl

Supplementary material file. DOI: 10.1107/S1600536812050866/lh5571Isup3.cml

Additional supplementary materials:  crystallographic information; 3D view; checkCIF report

## Figures and Tables

**Table 1:** Hydrogen-bond geometry (Å, °)

*D*—H⋯*A*	*D*—H	H⋯*A*	*D*⋯*A*	*D*—H⋯*A*
C10—H10⋯O1^i^	0.95	2.23	3.1575 (14)	166

Symmetry code: (i) 
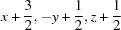
.

**Fig. 1.**
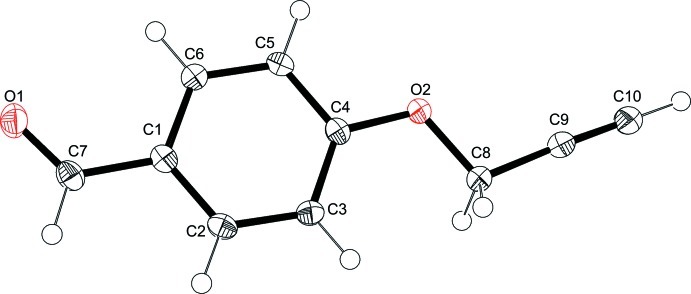
The molecular structure of the title compound with displacement ellipsoids drawn at the 50% probability level and H atoms are shown as small spheres.

**Fig. 2.**
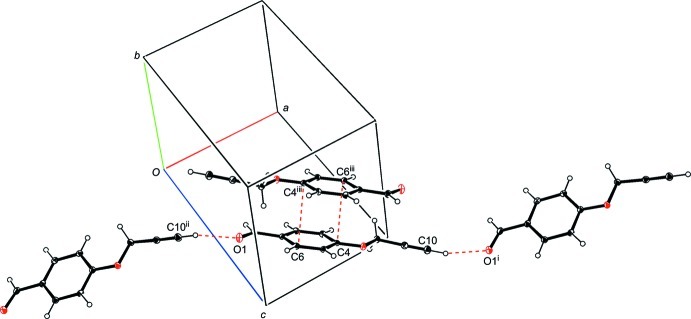
Part of the crystal structure showing the ladder-like array formed by π–π stacking interactions and weak C—H···O hydrogen bonds (dashed lines) [Symmetry codes: (i) x + 3/2, -y + 1/2, z + 1/2; (ii) x - 3/2, -y + 1/2, z - 1/2; (iii) -x + 1, -y + 1, -z + 2].

## supplementary crystallographic information

### Comment

The title compound, C_10_H_8_O_2_, is a benzaldehyde derivative whose structure is often observed in macrocyclic compounds (Berscheid *et al.* 1992; Mohr *et al.* 2003). Analogues of the title compound have already been reported as an ester (Nieger *et al.*, 2004), as a ketone (Ranjith *et al.*, 2010) and as an α,β-unsatuated ketone (Zhang *et al.*, 2011). The molecule has a planar structure (atoms C1—C10/O1—O2 are essentailly co-planar with an r.m.s. deviation = 0.0192 Å), indicating an effective conjugation of the carbonyl group, the C1—C6 benzene ring and the lone pair of atom O2.

The molecular structure of the title compound is shown in Fig. 1. In the crystal, molecules form dimers across inversion centers (Fig. 2), owing to π–π stacking interactions. The intermolecular distance of C4···C6^iii^ is 3.3026 (16) Å [Symmetry code:(iii) -*x* + 1, -*y* + 1, -*z* + 2.]. The molecules also form weak intermolecular C—H···O═C hydrogen bonds between the carbonyl oxygen and acetylene group to give a ladder-like structure where the distances of C10···O1^i^ and O1···C10^ii^ are 3.1575 (14) Å [Symmetry codes:(i) *x* + 3/2, -*y* + 1/2, *z* + 1/2 and (ii) *x* - 3/2, -*y* + 1/2, *z* - 1/2.].

### Experimental

A mixture of 4-hydroxybenzaldehyde (1.22 g, 10 mmol) and 3-bromoprop-1-yne (3.57 g, 30 mmol) in 1-methylpyrrolidin-2-one (20 ml) was heated at 473K for 2 h in the presence of K_2_CO_3_ (4.15 g). The solution was poured into water and extracted by benzene (100 ml). The organic layer was washed with 5% NaOHaq, 5% Na_2_CO_3_aq and water, and was dried over Na_2_SO_4_. After removal of Na_2_SO_4_ and benzene, the residue was recrystallized by hexane to give 0.96 g (60%) of the title compound as a pale yellow powder. Single crystals with sufficient quality were prepared by sublimation at room temperature.

### Refinement

The C-bound H atoms were placed at ideal positions and were refined as riding on their parent C atoms. *U*_iso_(H) values of the H atoms were set at 1.2*U*_eq_(parent atom).

### Crystal data

**Table:**

C_10_H_8_O_2_	*F*(000) = 336.00
*M**_r_* = 160.17	*D*_x_ = 1.327 Mg m^−^^3^
Monoclinic, *P*2_1_/*n*	Mo *K*α radiation, λ = 0.71075 Å
Hall symbol: -P 2yn	Cell parameters from 2598 reflections
*a* = 7.906 (3) Å	θ = 2.6–31.1°
*b* = 7.385 (2) Å	µ = 0.09 mm^−^^1^
*c* = 14.036 (5) Å	*T* = 93 K
β = 102.025 (5)°	Prism, colorless
*V* = 801.5 (5) Å^3^	0.20 × 0.10 × 0.10 mm
*Z* = 4	

### Data collection

**Table:**

Rigaku Saturn724+ diffractometer	1669 reflections with *F*^2^ > 2σ(*F*^2^)
Detector resolution: 7.111 pixels mm^-1^	*R*_int_ = 0.044
ω scans	θ_max_ = 27.5°
Absorption correction: numerical (*NUMABS*; Rigaku, 1999)	*h* = −10→10
*T*_min_ = 0.984, *T*_max_ = 0.991	*k* = −9→8
6309 measured reflections	*l* = −18→13
1832 independent reflections	

### Refinement

**Table:**

Refinement on *F*^2^	Secondary atom site location: difference Fourier map
*R*[*F*^2^ > 2σ(*F*^2^)] = 0.037	Hydrogen site location: inferred from neighbouring sites
*wR*(*F*^2^) = 0.101	H-atom parameters constrained
*S* = 1.06	*w* = 1/[σ^2^(*F*_o_^2^) + (0.0503*P*)^2^ + 0.2315*P*] where *P* = (*F*_o_^2^ + 2*F*_c_^2^)/3
1831 reflections	(Δ/σ)_max_ = 0.001
141 parameters	Δρ_max_ = 0.32 e Å^−^^3^
0 restraints	Δρ_min_ = −0.17 e Å^−^^3^
Primary atom site location: structure-invariant direct methods	

### Special details

**Table:**

Refinement. Refinement was performed using all reflections. except for one with very negative *F*^2^. The weighted *R*-factor (*wR*) and goodness of fit (*S*) are based on *F*^2^. *R*-factor (gt) are based on *F*. The threshold expression of *F*^2^ > 2.0 σ(*F*^2^) is used only for calculating *R*-factor (gt).

### Fractional atomic coordinates and isotropic or equivalent isotropic displacement parameters (Å^2^)

**Table:**

	*x*	*y*	*z*	*U*_iso_*/*U*_eq_	
O1	−0.02874 (9)	0.43434 (11)	0.86519 (6)	0.0269 (3)	
O2	0.72238 (8)	0.20161 (10)	1.09425 (5)	0.0184 (2)	
C1	0.26055 (12)	0.32435 (14)	0.90578 (7)	0.0169 (3)	
C2	0.39397 (13)	0.24978 (14)	0.86774 (7)	0.0183 (3)	
C3	0.55122 (13)	0.20458 (14)	0.92785 (7)	0.0176 (3)	
C4	0.57337 (12)	0.23630 (13)	1.02780 (7)	0.0160 (3)	
C5	0.43929 (12)	0.30938 (13)	1.06687 (7)	0.0167 (3)	
C6	0.28397 (12)	0.35345 (13)	1.00640 (7)	0.0171 (3)	
C7	0.09823 (13)	0.37510 (14)	0.83968 (7)	0.0206 (3)	
C8	0.86477 (12)	0.13260 (14)	1.05624 (7)	0.0185 (3)	
C9	1.00867 (12)	0.09585 (15)	1.13789 (7)	0.0198 (3)	
C10	1.12987 (14)	0.06479 (16)	1.20170 (8)	0.0242 (3)	
H2	0.3760	0.2314	0.7979	0.0271*	
H3	0.6428	0.1534	0.9001	0.0187*	
H5	0.4618	0.3309	1.1384	0.0247*	
H6	0.1910	0.4060	1.0336	0.0199*	
H7	0.1033	0.3559	0.7691	0.0267*	
H8A	0.8996	0.2240	1.0127	0.0191*	
H8B	0.8295	0.0199	1.0208	0.0222*	
H10	1.2291	0.0429	1.2516	0.0372*	

### Atomic displacement parameters (Å^2^)

**Table:**

	*U*^11^	*U*^22^	*U*^33^	*U*^12^	*U*^13^	*U*^23^
O1	0.0204 (4)	0.0323 (5)	0.0256 (5)	0.0041 (3)	−0.0007 (3)	−0.0039 (3)
O2	0.0168 (4)	0.0228 (4)	0.0150 (4)	0.0031 (3)	0.0020 (3)	−0.0004 (3)
C1	0.0187 (5)	0.0144 (5)	0.0168 (5)	−0.0028 (4)	0.0016 (4)	0.0003 (4)
C2	0.0219 (5)	0.0178 (5)	0.0149 (5)	−0.0030 (4)	0.0030 (4)	−0.0011 (4)
C3	0.0194 (5)	0.0172 (5)	0.0169 (5)	−0.0003 (4)	0.0051 (4)	−0.0014 (4)
C4	0.0174 (5)	0.0134 (5)	0.0162 (5)	−0.0015 (4)	0.0015 (4)	0.0013 (4)
C5	0.0197 (5)	0.0167 (5)	0.0138 (5)	−0.0015 (4)	0.0034 (4)	0.0004 (4)
C6	0.0177 (5)	0.0158 (5)	0.0180 (5)	−0.0007 (4)	0.0040 (4)	−0.0003 (4)
C7	0.0214 (5)	0.0203 (5)	0.0182 (5)	−0.0019 (4)	−0.0004 (4)	−0.0022 (4)
C8	0.0179 (5)	0.0202 (5)	0.0177 (5)	0.0025 (4)	0.0041 (4)	−0.0002 (4)
C9	0.0196 (5)	0.0207 (5)	0.0199 (5)	0.0007 (4)	0.0060 (4)	−0.0011 (4)
C10	0.0213 (5)	0.0303 (6)	0.0210 (5)	0.0026 (5)	0.0045 (4)	−0.0001 (5)

### Geometric parameters (Å, º)

**Table:**

O1—C7	1.2154 (14)	C8—C9	1.4622 (13)
O2—C4	1.3659 (11)	C9—C10	1.1897 (14)
O2—C8	1.4360 (13)	C2—H2	0.970
C1—C2	1.3913 (16)	C3—H3	0.968
C1—C6	1.4021 (15)	C5—H5	0.995
C1—C7	1.4670 (14)	C6—H6	0.977
C2—C3	1.3899 (14)	C7—H7	1.010
C3—C4	1.3968 (15)	C8—H8A	0.988
C4—C5	1.3993 (15)	C8—H8B	0.980
C5—C6	1.3784 (13)	C10—H10	0.950
			
O1···C6	2.8906 (13)	C7···H8B^iv^	3.4936
O2···C10	3.4132 (15)	C7···H10^iii^	2.9850
C1···C4	2.7763 (14)	C8···H2^viii^	3.5206
C2···C5	2.7778 (17)	C8···H6^xiii^	3.3423
C3···C6	2.8035 (17)	C8···H8A^vi^	3.4770
C3···C8	2.7953 (15)	C8···H8B^vi^	3.0613
O1···O2^i^	3.5839 (13)	C9···H2^viii^	2.9585
O1···C6^ii^	3.3603 (15)	C9···H3^vi^	3.4464
O1···C8^i^	3.5397 (16)	C9···H6^xiii^	3.2166
O1···C9^i^	3.4739 (17)	C9···H8A^vi^	3.3479
O1···C10^iii^	3.1575 (14)	C9···H8B^vi^	2.9185
O2···O1^i^	3.5839 (13)	C10···H2^viii^	3.0407
O2···C1^i^	3.5034 (17)	C10···H3^vi^	2.9896
O2···C2^iv^	3.5282 (16)	C10···H3^viii^	3.4607
O2···C6^i^	3.5729 (15)	C10···H5^xiii^	3.5367
O2···C7^i^	3.4779 (15)	C10···H5^xiv^	3.0372
C1···O2^i^	3.5034 (17)	C10···H6^xiii^	3.5534
C1···C4^i^	3.5512 (17)	C10···H8B^vi^	3.2660
C1···C5^i^	3.5660 (16)	H2···O1^xi^	3.5583
C1···C8^iv^	3.5880 (18)	H2···O2^v^	2.9057
C2···O2^iv^	3.5282 (16)	H2···C7^xi^	3.2950
C2···C5^i^	3.5602 (17)	H2···C8^v^	3.5206
C2···C10^v^	3.5491 (18)	H2···C9^v^	2.9585
C3···C4^iv^	3.4949 (17)	H2···C10^v^	3.0407
C3···C5^i^	3.5907 (18)	H2···H5^i^	3.5239
C3···C6^i^	3.5640 (17)	H2···H5^v^	3.5908
C4···C1^i^	3.5512 (17)	H2···H7^xi^	2.9435
C4···C3^iv^	3.4949 (17)	H2···H10^v^	3.4275
C4···C6^i^	3.3026 (16)	H3···O1^xiii^	3.4379
C5···C1^i^	3.5660 (16)	H3···C4^iv^	3.5984
C5···C2^i^	3.5602 (17)	H3···C5^iv^	3.5273
C5···C3^i^	3.5907 (18)	H3···C9^vi^	3.4464
C6···O1^ii^	3.3603 (15)	H3···C10^vi^	2.9896
C6···O2^i^	3.5729 (15)	H3···C10^v^	3.4607
C6···C3^i^	3.5640 (17)	H3···H6^i^	3.5598
C6···C4^i^	3.3026 (16)	H3···H7^xi^	3.5107
C7···O2^i^	3.4779 (15)	H3···H10^vi^	2.9257
C8···O1^i^	3.5397 (16)	H3···H10^v^	3.2297
C8···C1^iv^	3.5880 (18)	H5···C1^i^	3.5007
C8···C8^vi^	3.5082 (17)	H5···C2^i^	3.3077
C8···C9^vi^	3.5225 (17)	H5···C3^i^	3.5504
C9···O1^i^	3.4739 (17)	H5···C7^viii^	3.1921
C9···C8^vi^	3.5225 (17)	H5···C10^x^	3.5367
C10···O1^vii^	3.1575 (14)	H5···C10^xii^	3.0372
C10···C2^viii^	3.5491 (18)	H5···H2^i^	3.5239
O1···H6	2.6337	H5···H2^viii^	3.5908
O2···H3	2.6899	H5···H7^viii^	2.3799
O2···H5	2.4642	H5···H10^x^	3.4131
C1···H3	3.2914	H5···H10^xii^	2.8432
C1···H5	3.3209	H6···O1^ii^	2.4110
C2···H6	3.2988	H6···O2^i^	3.5493
C2···H7	2.5468	H6···C3^i^	3.5012
C3···H5	3.3126	H6···C4^i^	3.4439
C3···H8A	2.7677	H6···C7^ii^	3.5637
C3···H8B	2.6843	H6···C8^x^	3.3423
C4···H2	3.2789	H6···C9^x^	3.2166
C4···H6	3.2895	H6···C10^x^	3.5534
C4···H8A	2.6337	H6···H3^i^	3.5598
C4···H8B	2.5972	H6···H6^ii^	3.2783
C5···H3	3.3114	H6···H8A^x^	2.6299
C6···H2	3.2855	H6···H8A^i^	2.8648
C6···H7	3.3396	H6···H8B^iv^	3.2330
C7···H2	2.6135	H6···H10^xii^	3.1175
C7···H6	2.6767	H7···O2^v^	2.8373
C8···H3	2.5110	H7···C2^ix^	3.4881
C10···H8A	3.1180	H7···C4^v^	3.4138
C10···H8B	3.1119	H7···C5^v^	3.1133
H2···H3	2.3616	H7···H2^ix^	2.9435
H2···H7	2.3008	H7···H3^ix^	3.5107
H3···H8A	2.3572	H7···H5^v^	2.3799
H3···H8B	2.2315	H7···H10^iii^	3.0118
H5···H6	2.3967	H8A···O1^xiii^	2.7399
O1···H2^ix^	3.5583	H8A···O1^i^	3.1014
O1···H3^x^	3.4379	H8A···C1^xiii^	3.5673
O1···H6^ii^	2.4110	H8A···C6^xiii^	3.2041
O1···H8A^x^	2.7399	H8A···C6^i^	3.4287
O1···H8A^i^	3.1014	H8A···C7^xiii^	3.3458
O1···H10^iii^	2.2281	H8A···C8^vi^	3.4770
O2···H2^viii^	2.9057	H8A···C9^vi^	3.3479
O2···H6^i^	3.5493	H8A···H6^xiii^	2.6299
O2···H7^viii^	2.8373	H8A···H6^i^	2.8648
C1···H5^i^	3.5007	H8A···H8B^vi^	2.9105
C1···H8A^x^	3.5673	H8B···C1^iv^	2.8875
C1···H8B^iv^	2.8875	H8B···C2^iv^	3.2690
C2···H5^i^	3.3077	H8B···C5^iv^	3.2946
C2···H7^xi^	3.4881	H8B···C6^iv^	2.9001
C2···H8B^iv^	3.2690	H8B···C7^iv^	3.4936
C3···H5^i^	3.5504	H8B···C8^vi^	3.0613
C3···H6^i^	3.5012	H8B···C9^vi^	2.9185
C4···H3^iv^	3.5984	H8B···C10^vi^	3.2660
C4···H6^i^	3.4439	H8B···H6^iv^	3.2330
C4···H7^viii^	3.4138	H8B···H8A^vi^	2.9105
C5···H3^iv^	3.5273	H8B···H8B^vi^	2.8905
C5···H7^viii^	3.1133	H10···O1^vii^	2.2281
C5···H8B^iv^	3.2946	H10···C5^xiv^	3.5569
C5···H10^xii^	3.5569	H10···C7^vii^	2.9850
C6···H8A^x^	3.2041	H10···H2^viii^	3.4275
C6···H8A^i^	3.4287	H10···H3^vi^	2.9257
C6···H8B^iv^	2.9001	H10···H3^viii^	3.2297
C7···H2^ix^	3.2950	H10···H5^xiii^	3.4131
C7···H5^v^	3.1921	H10···H5^xiv^	2.8432
C7···H6^ii^	3.5637	H10···H6^xiv^	3.1175
C7···H8A^x^	3.3458	H10···H7^vii^	3.0118
			
C4—O2—C8	116.38 (8)	C3—C2—H2	120.247
C2—C1—C6	119.71 (9)	C2—C3—H3	119.929
C2—C1—C7	119.45 (9)	C4—C3—H3	121.483
C6—C1—C7	120.82 (10)	C4—C5—H5	117.787
C1—C2—C3	121.06 (10)	C6—C5—H5	122.175
C2—C3—C4	118.59 (10)	C1—C6—H6	120.143
O2—C4—C3	124.34 (10)	C5—C6—H6	120.002
O2—C4—C5	114.89 (9)	O1—C7—H7	122.939
C3—C4—C5	120.77 (9)	C1—C7—H7	112.094
C4—C5—C6	120.02 (10)	O2—C8—H8A	109.216
C1—C6—C5	119.85 (10)	O2—C8—H8B	109.121
O1—C7—C1	124.97 (10)	C9—C8—H8A	109.879
O2—C8—C9	108.48 (9)	C9—C8—H8B	109.452
C8—C9—C10	177.37 (12)	H8A—C8—H8B	110.659
C1—C2—H2	118.687	C9—C10—H10	177.936
			
C4—O2—C8—C9	−177.31 (7)	C7—C1—C6—C5	−178.20 (9)
C8—O2—C4—C3	1.73 (13)	C1—C2—C3—C4	−0.28 (15)
C8—O2—C4—C5	−177.84 (8)	C2—C3—C4—O2	−178.56 (9)
C2—C1—C6—C5	0.50 (15)	C2—C3—C4—C5	0.99 (15)
C6—C1—C2—C3	−0.46 (15)	O2—C4—C5—C6	178.63 (8)
C2—C1—C7—O1	176.92 (10)	C3—C4—C5—C6	−0.96 (14)
C7—C1—C2—C3	178.26 (9)	C4—C5—C6—C1	0.20 (14)
C6—C1—C7—O1	−4.38 (16)		

Symmetry codes: (i) −*x*+1, −*y*+1, −*z*+2; (ii) −*x*, −*y*+1, −*z*+2; (iii) *x*−3/2, −*y*+1/2, *z*−1/2; (iv) −*x*+1, −*y*, −*z*+2; (v) *x*−1/2, −*y*+1/2, *z*−1/2; (vi) −*x*+2, −*y*, −*z*+2; (vii) *x*+3/2, −*y*+1/2, *z*+1/2; (viii) *x*+1/2, −*y*+1/2, *z*+1/2; (ix) −*x*+1/2, *y*+1/2, −*z*+3/2; (x) *x*−1, *y*, *z*; (xi) −*x*+1/2, *y*−1/2, −*z*+3/2; (xii) −*x*+3/2, *y*+1/2, −*z*+5/2; (xiii) *x*+1, *y*, *z*; (xiv) −*x*+3/2, *y*−1/2, −*z*+5/2.

### Hydrogen-bond geometry (Å, º)

**Table:**

*D*—H···*A*	*D*—H	H···*A*	*D*···*A*	*D*—H···*A*
C10—H10···O1^vii^	0.95	2.23	3.1575 (14)	166

Symmetry code: (vii) *x*+3/2, −*y*+1/2, *z*+1/2.
